# Serum and Glucocorticoid-Inducible Kinase1 Increases Plasma Membrane wt-CFTR in Human Airway Epithelial Cells by Inhibiting Its Endocytic Retrieval

**DOI:** 10.1371/journal.pone.0089599

**Published:** 2014-02-21

**Authors:** Jennifer M. Bomberger, Bonita A. Coutermarsh, Roxanna L. Barnaby, J. Denry Sato, M. Christine Chapline, Bruce A. Stanton

**Affiliations:** 1 Department of Microbiology and Molecular Genetics, University of Pittsburgh School of Medicine, Pittsburgh, Pennsylvania, United States of America; 2 Department of Microbiology and Immunology and of Physiology, The Geisel School of Medicine at Dartmouth, Hanover, New Hampshire, United States of America; 3 Mt. Desert Island Biological Laboratory, Salisbury Cove, Maine, United States of America; Emory University School of Medicine, United States of America

## Abstract

**Background:**

Chloride (Cl) secretion by the Cystic Fibrosis Transmembrane Conductance Regulator (CFTR) located in the apical membrane of respiratory epithelial cells plays a critical role in maintenance of the airway surface liquid and mucociliary clearance of pathogens. Previously, we and others have shown that the serum and glucocorticoid-inducible kinase-1 (SGK1) increases wild type CFTR (wt-CFTR) mediated Cl transport in *Xenopus* oocytes by increasing the amount of wt-CFTR protein in the plasma membrane. However, the effect of SGK1 on the membrane abundance of wt-CFTR in airway epithelial cells has not been examined, and the mechanism whereby SGK1 increases membrane wt-CFTR has also not been examined. Thus, the goal of this study was to elucidate the mechanism whereby SGK1 regulates the membrane abundance of wt-CFTR in human airway epithelial cells.

**Methods and Results:**

We report that elevated levels of SGK1, induced by dexamethasone, increase plasma membrane abundance of wt-CFTR. Reduction of SGK1 expression by siRNA (siSGK1) and inhibition of SGK1 activity by the SGK inhibitor GSK 650394 abrogated the ability of dexamethasone to increase plasma membrane wt-CFTR. Overexpression of a constitutively active SGK1 (SGK1-S422D) increased plasma membrane abundance of wt-CFTR. To understand the mechanism whereby SGK1 increased plasma membrane wt-CFTR, we examined the effects of siSGK1 and SGK1-S442D on the endocytic retrieval of wt-CFTR. While siSGK1 increased wt-CFTR endocytosis, SGK1-S442D inhibited CFTR endocytosis. Neither siSGK1 nor SGK1-S442D altered the recycling of endocytosed wt-CFTR back to the plasma membrane. By contrast, SGK1 increased the endocytosis of the epidermal growth factor receptor (EGFR).

**Conclusion:**

This study demonstrates for the first time that SGK1 selectively increases wt-CFTR in the plasma membrane of human airway epithelia cells by inhibiting its endocytic retrieval from the membrane.

## Introduction

The Cystic Fibrosis Transmembrane Conductance Regulator (CFTR) is a cyclic-AMP regulated chloride (Cl) channel residing in the apical plasma membrane of a variety of epithelial cells, including those in the lungs [1]. Cl secretion via wt-CFTR is critical in maintaining the airway surface liquid volume, and therefore, is essential for effective mucociliary clearance, which mechanically clears debris and pathogens from the airways and serves a vital role in innate immunity [2]. Individuals with defective CFTR function, for example patients with Cystic Fibrosis or chronic smokers, have chronic lung infections due to a lack of clearance of respiratory pathogens [2], [3]. wt-CFTR function and localization in the cell are highly regulated by a complex of proteins that modulates its biosynthesis, trafficking to and from the plasma membrane, and channel activity [1], [4]–[7]. Once delivered to the apical plasma membrane of airway epithelial cells, wt-CFTR is rapidly endocytosed from the plasma membrane and then undergoes rapid and efficient recycling back to the plasma membrane [1], [8]–[11]. We and others have demonstrated that the endocytic trafficking of wt-CFTR is regulated by numerous protein-protein interactions, as well as by ubiquitination [1], [4]–[7], [12].

SGK1 is transcriptionally regulated by a variety of stimuli including stress, serum, and a large number of cytokines and hormones including gluococorticoids [13]. SGK1 regulates a number of ion channels including ENaC, CFTR, and ROMK that play important roles in sodium and potassium excretion by the kidneys, as well as in the maintenance of airway surface liquid volume in the lungs [5], [13]. A variant of SGK1 is associated with increased blood pressure, obesity and type II diabetes [13]. Previously, others and we have shown that SGK1 stimulates wt-CFTR mediated chloride (Cl) currents in *Xenopus* oocytes by increasing the amount of wt-CFTR protein in the plasma membrane [14], [15]. Moreover, SGK1 also increases the plasma membrane abundance of wt-CFTR in the plasma membrane of mitochondrion rich cells in the gill of *Fundulus heteroclitus* [16], [17]. In a recent study Caohuy et al [18] demonstrated that dexamethasone increases the plasma membrane expression of both wt-CFTR and ΔF508-CFTR in pancreatic cells, and that RNA interference of SGK1 blocks the stimulatory effect of dexamethasone on plasma membrane ΔF508-CFTR, a finding suggesting that SGK1 increases plasma membrane CFTR. In addition, two recent studies [19], [20] have shown that dexamethasone increases wt-CFTR abundance in airway cell lines. However, the mechanism whereby SGK1 increased the plasma membrane abundance of wt-CFTR has not been examined. Thus, the primary goal of this study was to elucidate the cellular mechanism whereby SGK1 increases plasma membrane wt-CFTR. The data herein is novel because it demonstrates that SGK1 selectively increases wt-CFTR in the plasma membrane of human airway epithelial cells by inhibiting its endocytic retrieval from the membrane.

## Materials and Methods

### Cell Culture

The role of SGK1 in regulating plasma membrane wt-CFTR was studied in human airway epithelial cells (CFBE41o- cells, homozygous for the ΔF508 mutation) stably expressing wt-CFTR, (generously provided by Dr. J.P. Clancy, University of Cincinnati)[21]. Details on the stable transfection and characterization of CFBE41o- cells expressing wt-CFTR (hereafter called CFBE cells) have been described in detail previously [10], [11]. Cells between passages 18 and 27 were grown in an air-liquid interface culture at 37°C [10], [11]. To establish confluent, polarized monolayers, 1×10^6^ cells were seeded onto 24-mm Transwell permeable supports (0.4-µm pore size, Corning, Corning, NY) coated with Vitrogen plating medium containing human fibronectin (10 µg/ml, Collaborative Biomedical Products, Bedford, MA), PureCol (1%, Inamed Biomaterials, Fremont, CA), and bovine serum albumin (10 µg/ml, Invitrogen) and grown in an air-liquid interface culture at 37°C for 6–9 days, as described [10], [11].

### RNA-mediated Interference

SGK1 expression was selectively reduced using siRNA purchased from Qiagen (Valencia, CA), using methods described previously [11]. Sequences for siSGK1 are: sense 5′ CAG CUG AAA UGU ACG ACA A 3′; antisense 5′ UUG UCG UAC AUU UCA GCU G 3′; and siNegative scrambled sense 5′ UUC UCC GAA CGU GUC ACG U 3′ and antisense 5′ ACG UGA CAC GUU CGG AGA A 3′.

### Biochemical Determination of the Apical Membrane wt-CFTR

The biochemical determination of apical membrane wt-CFTR was performed by domain selective cell membrane protein biotinylation using EZ-Link™ Sulfo-NHS-LC-Biotin (Pierce), as described previously in detail [22].

### wt-CFTR Endocytic and Recycling Assays

wt-CFTR and EGFR endocytosis and wt-CFTR recycling were measured in polarized monolayers of cells using a cell membrane protein biotinylation approach as described in detail by our laboratory [22]. In brief, for both assays, plasma membrane proteins were biotinylated at 4°C using EZ-Link™ Sulfo-NHS-SS-Biotin (Thermo Scientific Rockford, IL). For the endocytic assay, cells were then warmed to 37°C for 5 minutes, and then GSH was added at 4°C to reduce the disulfide bonds between proteins labeled with Sulfo-NHS-SS-Biotin in the plasma membrane. Biotinylated proteins that were endocytosed during the 5-minute period at 37°C are not reduced by GSH, which is impermeant to the plasma membrane, and therefore at this time in the protocol biotinylated proteins are only in endosomes. Thereafter, cells were lysed, and biotinylated proteins were isolated using streptavidin-agarose beads, eluted into SDS sample buffer, separated by 7.5% SDS-PAGE, and wt-CFTR and EGFR were detected by Western blot. The amount of biotinylated wt-CFTR and EGFR remaining in the plasma membrane after GSH treatment at 4°C and without the 37°C warming was considered background (<5% compared with the amount of biotinylated wt-CFTR and EGFR at 4°C without GSH treatment) and was subtracted from biotinylated wt-CFTR and EGFR after warming to 37°C. wt-CFTR and EGFR endocytosis were calculated after subtraction of the background and expressed as the percentage of biotinylated wt-CFTR and EGFR after warming to 37°C compared with the amount of biotinylated wt-CFTR and EGFR present before warming to 37°C (minus background). For the wt-CFTR endocytic recycling assay, cells were warmed to 37°C for 5 minutes after biotinylation to load endocytic vesicles with biotinylated proteins. Cells were then cooled to 4°C, and the disulfide bonds on Sulfo-NHS-SS-Biotin-labeled proteins remaining in the plasma membrane were reduced by GSH at 4°C. Subsequently, cells were either lysed (to detect the amount of wt-CFTR in endosomes) or warmed again to 37°C for 5 minutes (to allow biotinylated wt-CFTR to recycle from endosomes back to the plasma membrane). Cells were then cooled again to 4°C, and the disulfide bonds on the proteins bound to Sulfo-NHS-SS-Biotin remaining in the plasma membrane were reduced with GSH. The recycling of endocytosed wt-CFTR from endosomes to the apical plasma membrane was calculated as the difference between the amount of biotinylated wt-CFTR after the first and second GSH treatments.

### Western Blot Analysis

Western blot analysis of SGK1, wt-CFTR, ezrin, EEA1, Rab5a, Rab11a and EGFR were conducted by methods described previously [4], [22].

### Intracellular Vesicle Isolation

To examine the effect of SGK1 on the endosomal trafficking of wt-CFTR, differential centrifugation combined with immunoprecipitation techniques were used to isolate early and recycling endosomes from airway epithelial cells, as described previously by our laboratory and originally described by Barile *et al* [22]–[24]. In brief, CFBE cells with or without SGK1 knockdown, by siSGK1, were scraped from filters into isolation buffer (10 mM triethanolamine, 250 mM sucrose, pH to 7.6, 8 mg/liter PMSF and 0.08 mg/liter leupeptin). Cells were homogenized with plastic tube homogenizer and centrifuged at 4000×g for 10 minutes at 4°C. Supernatant was saved, homogenization repeated on the pellet. Pooled supernatants were centrifuged at 17000×g for 20 minutes at 4°C, then supernatant centrifuged at 200,000×g for one hour at 4°C. The pellet was resuspended via homogenization and centrifuged a second time for one hour at 200,000×g at 4°C. The pellet was resuspended in PBS containing protease inhibitors and wt-CFTR was immunprecipitated from this fraction (intracellular vesicle fraction). Control experiments were performed with a non-specific antibody (IgG). Immunoprecipitated proteins, including wt-CFTR and proteins associated with wt-CFTR-containing vesicles, were run on an SDS PAGE gel whereupon western blots were performed using antibodies to either Rab 5a (to determine the amount of wt-CFTR in early endosomes), EEA1 (to determine the amount of wt-CFTR in early endosomes), and Rab 11a (to determine the amount of wt-CFTR in recycling endosomes).

### RNA Isolation and Quantitative-Reverse Transcription-PCR

Quantitative*-*Reverse transcription (Q-RT-PCR) studies were conducted to examine the effect of dexamethasone (50 nM) on the expression of SGK1 mRNA in CFBE cells, as previously described in detail [16]. In brief, total RNA was isolated from cells treated with vehicle (0.01% ethanol in PBS, i.e., control) or dexamethasone using the RNAeasy Mini Kit (Qiagen, Valencia, CA). RNA was treated with DNase (DNA-Free, Ambion, Austin, TX) to remove contaminating DNA. Total RNA was quantified using spectrophotometric OD260/280 measurements (NanoDrop, NanoDrop Technologies, Rockland, DE) and RNA quality was assessed with an Agilent 2100 Bioanalyzer (Agilent Technologies, Wilmington, DE). A Taqman Gene Expression assay was used for Q-RT-PCR for human SGK1 (Applied Biosystems, Foster City, CA catalog # 4331182). Q-RT-PCR products were run on a low melting point agarose gel to confirm amplicon size, subcloned into pCR4-TOPO (Invitrogen), and submitted for sequence analysis to confirm the identity of the product.

### Antibodies and Reagents

The antibodies used were: rabbit anti-SGK1 antibody (Sigma); mouse anti-CFTR C-terminus antibody (clone 24-1; R&D systems, Minneapolis, MN); mouse anti-CFTR antibody (clone 596; University of North Carolina Cystic Fibrosis Center, Chapel Hill, NC); mouse anti-ezrin, mouse anti-Rab5a and mouse anti-EEA1 antibodies (BD Biosciences, San Jose, CA); rabbit anti-Rab 11a (Life Technologies Corp); mouse anti-EGFR (Santa Cruz Biotechnology, Inc, Santa Cruz, CA); horseradish peroxidase-conjugated goat anti-mouse and goat anti-rabbit secondary antibodies (Bio-Rad, Hercules, CA). SGK1-S442D, a constitutively active form of human SGK1 and human SGK1-K127N, an inactive form of human SGK1, were constructed as described [15]. The SGK inhibitor GSK 650394 was obtained from Tocris (purchased through R&D Systems, Minneapolis, MN). All antibodies and reagents were used at the concentrations recommended by the manufacturers.

### Data Analysis and Statistics

Graphpad Prism version 5.0 for Macintosh (Graphpad, San Diego, CA) was used to perform a statistical analysis of the data. Means were compared using a t-test or ANOVA followed by Tukeys test, as appropriate. P<0.05 was significant, and all data are expressed as the mean ± SEM.

## Results

### Dexamethasone Increases SGK1 mRNA and Protein Abundance

**Figure 1:**
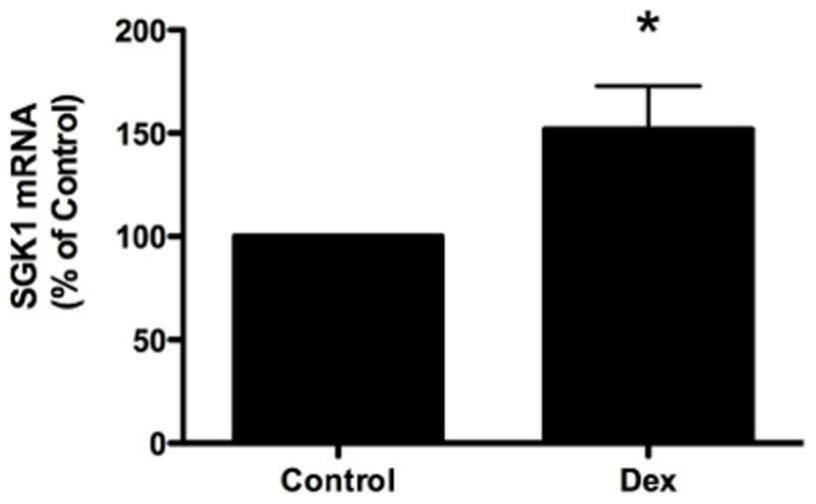
Dexamethasone increased SGK1 mRNA expression in CFBE cells. Dexamethasone (50 nM for 30 minutes) increased SGK1 mRNA versus control, as determined by Q-RT-PCR. Experiments performed three times. *p<0.05.

**Figure 2:**
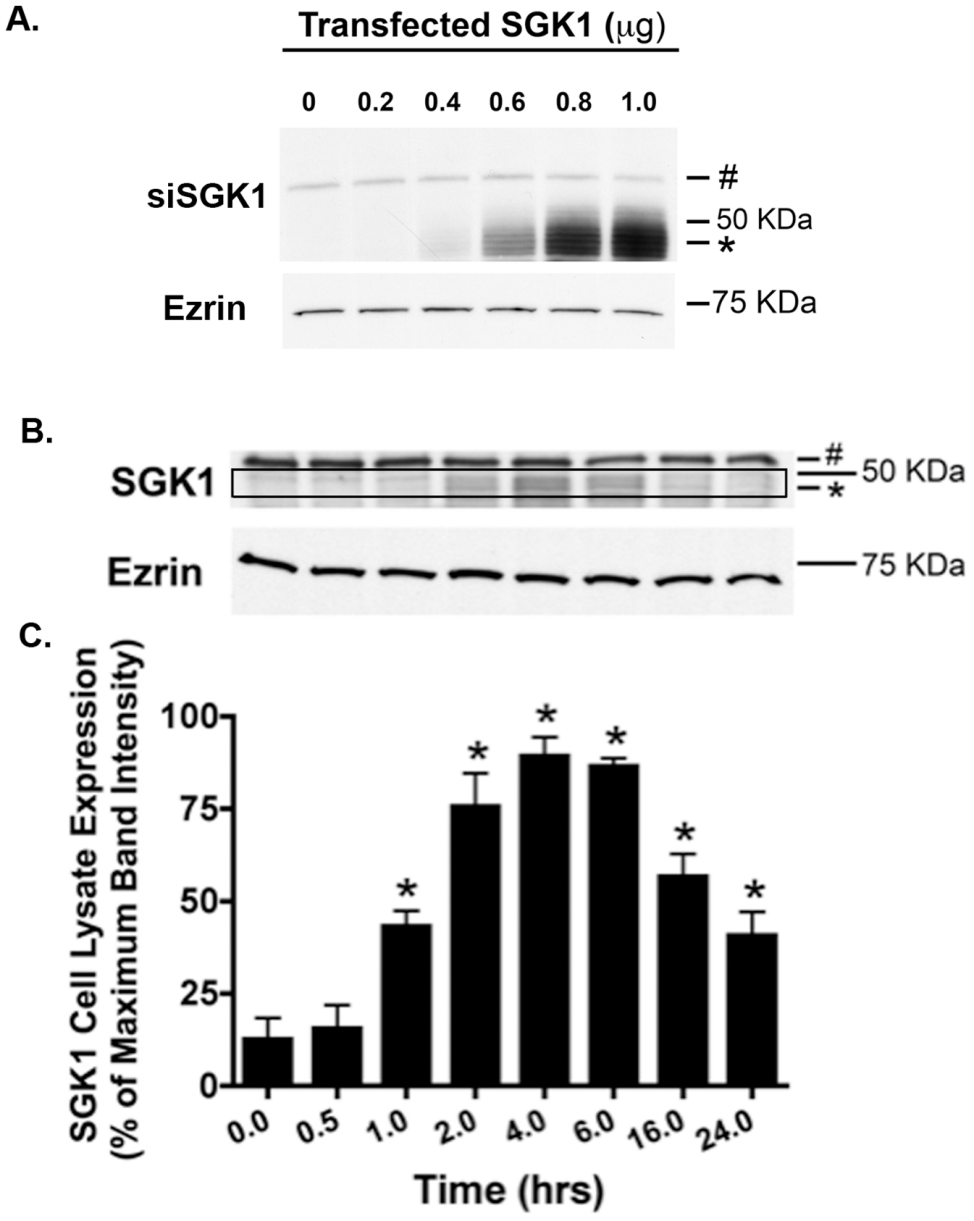
Dexamethasone increased SGK1 protein abundance in a time-dependent manner. (A) Western blot demonstrating that the Sigma SGK1 antibody recognizes SGK1. CFBE cells were transfected with cDNA (0, no SGK1 insert), or SGK1 cDNA (0.2, 0.4, 0.6, 0.8 and 1.0 µg) and 48 hours later cells were lysed and SGK1 and ezrin were analyzed by Western blot. The SGK1 antibody recognized a protein (∼48 kDa, the predicted molecular mass of SGK1) that increases in intensity as the amount of SGK1 cDNA transfected into CFBE cells was increased. By contrast, the intensity of a non-specific protein (>50 kDa, marked by a # sign in Figure 2A), did not change as a function of the amount of SGK1 cDNA transfected in CFBE cells. (B) Representative western blot of an experiment in which CFBE cells were treated with dexamethasone (50 nM) for the indicated times at 37°C. Subsequently, cells were lysed and SGK1 and ezrin were analyzed by western blot. Data expressed as a percent of the maximum SGK1 levels observed at 4 hours (100%). Ezrin was monitored as a loading control (each lane was loaded with 50 µg of protein). # indicates a non-specific protein detected by the SGK1 antibody. The black box highlights SGK1 protein, which is also indicated by an *. (C) Summary of three experiments. Data expressed as SGK1 normalized for the amount of ezrin. *p<0.05 vs. 0 time point (no dexamethasone treatment: cells were treated with vehicle).

Stress and glucocorticoids are well known stimuli of SGK1 gene expression [13]. To determine if dexamethasone increases SGK1 expression in human airway epithelial cells, CFBE cells were treated with vehicle (control) or dexamethasone (50 nM for 30 minutes) and SGK1 mRNA was measured by Q-RT-PCR. Dexamethasone significantly increased SGK1 mRNA compared to control (Figure 1). To determine if dexamethasone also increased SGK1 protein abundance we conducted western blot analysis of CFBE cells treated with dexamethasone or vehicle. First, we conducted a western blot analysis of CFBE cells transfected with several concentrations of SGK1 cDNA to verify that the SGK1 antibody recognizes human SGK1. Figure 2A reveals that the SGK1 antibody recognizes a protein (∼48 kDa, the molecular mass of SGK1) that increases in intensity as the amount of SGK1 cDNA transfected into CFBE cells was increased. By contrast, the intensity of a non-specific protein (>50 kDa, marked by a # sign in Figure 2A) did not change as a function of the amount of SGK1 cDNA transfected. Using the SGK1 antibody we observed that dexamethasone rapidly, and significantly, increased SGK1 protein abundance (Figures 2B and 2C). Although SGK1 levels were low in control cells, an increase in SGK1 protein was observed after 1 hour, peaked 4 hours after dexamethasone exposure and remained elevated for the duration of the experiment compared to vehicle treated CFBE cells (time = 0, Figures 2B and 2C).

### Dexamethasone Increases Plasma Membrane wt-CFTR

**Figure 3:**
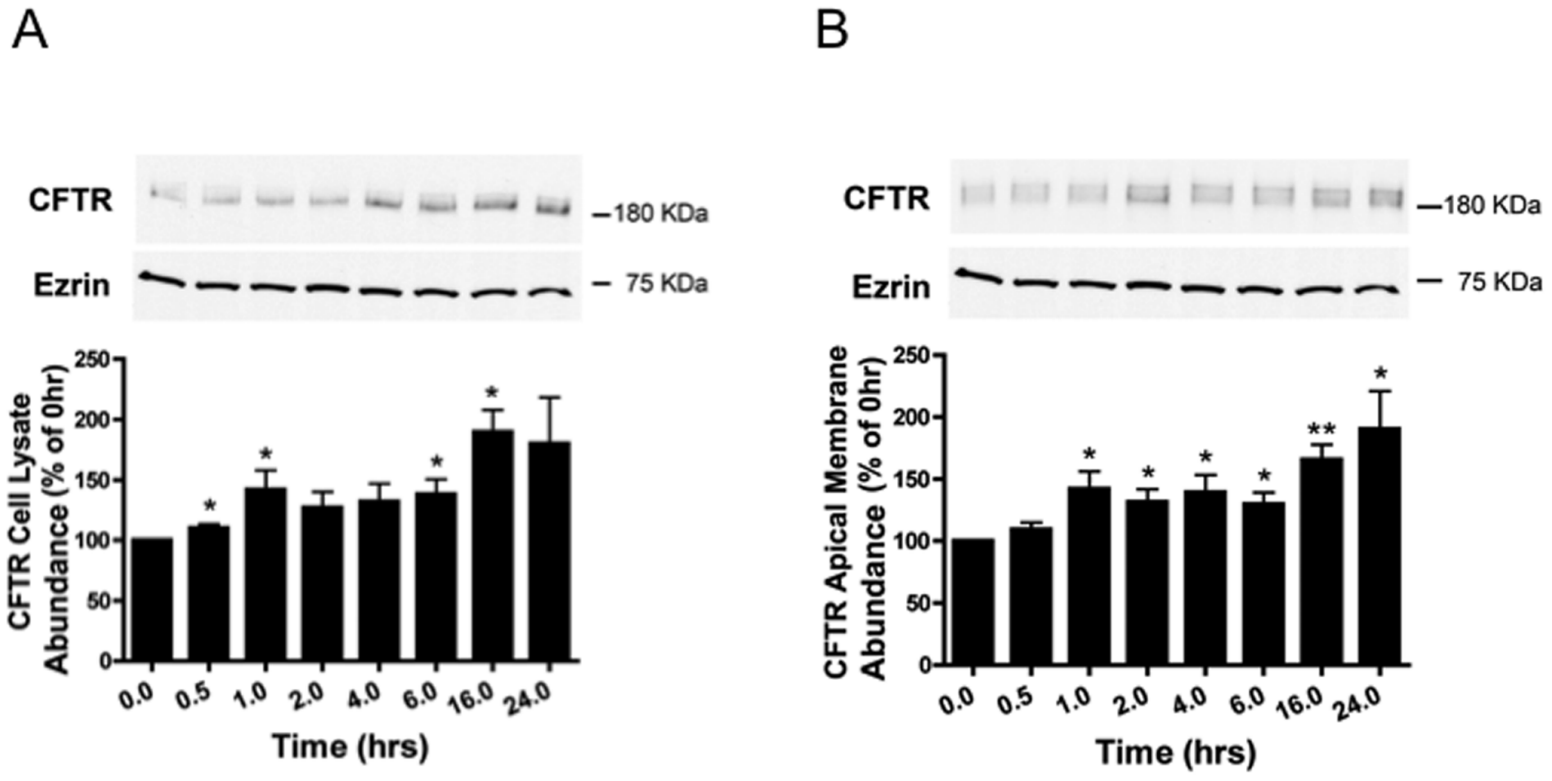
Dexamethasone increased wt-CFTR abundance in cell lysates (A), and in the apical membrane (B). Representative western blots and data summaries are presented. (A) Cells were treated with dexamethasone (50 nM) for the indicated times at 37°C and western blot analysis was conducted to measure wt-CFTR and ezrin in cell lysates. The molecular mass of wt-CFTR is ∼180 kDa. Ezrin was monitored as a loading control. n = 7 per time point. *p<0.05 versus 0 time point. (B) Cells were treated with dexamethasone (50 nM) for the indicated time points at 37°C, and subsequently apical plasma membrane proteins were biotinylated using a technique previously described in detail by our laboratory [12], [22]. Briefly, apical membrane proteins were biotinylated at 4°C using EZ-Link™ Sulfo-NHS-SS-Biotin. Subsequently, cells were lysed, and the biotinylated proteins were isolated by streptavidin-agarose beads, eluted into SDS sample buffer, and separated by 7.5% SDS-PAGE. The blots were probed for wt-CFTR (∼180 kDa) and for ezrin, a cytoplasmic protein that was not present in the biotinylated samples, confirming that biotin is impermeable to cell membranes [12], [22], and that the procedure to determine apical membrane wt-CFTR does not detect intracellular wt-CFTR. In this and all subsequent biotinylation experiments ezrin was never detected in the biotinylated samples by western blot, demonstrating that only plasma membrane proteins were biotinylated. *p<0.05 versus 0 time point. **p<0.01 versus 0 time point.

We also examined the effect of dexamethasone on wt-CFTR abundance in cell lysates, as well as in the apical membrane using cell-surface protein biotinylation and Western blot analysis. Dexamethasone rapidly, and significantly, increased wt-CFTR abundance in the cell lysate (Figure 3A) and in the apical plasma membrane (Figure 3B). The increase in apical plasma membrane wt-CFTR was significantly increased one hour after dexamethasone exposure and persisted for the duration of the experiment (24 hours).

### SGK1 Increases Apical Membrane wt-CFTR Abundance

**Figure 4:**
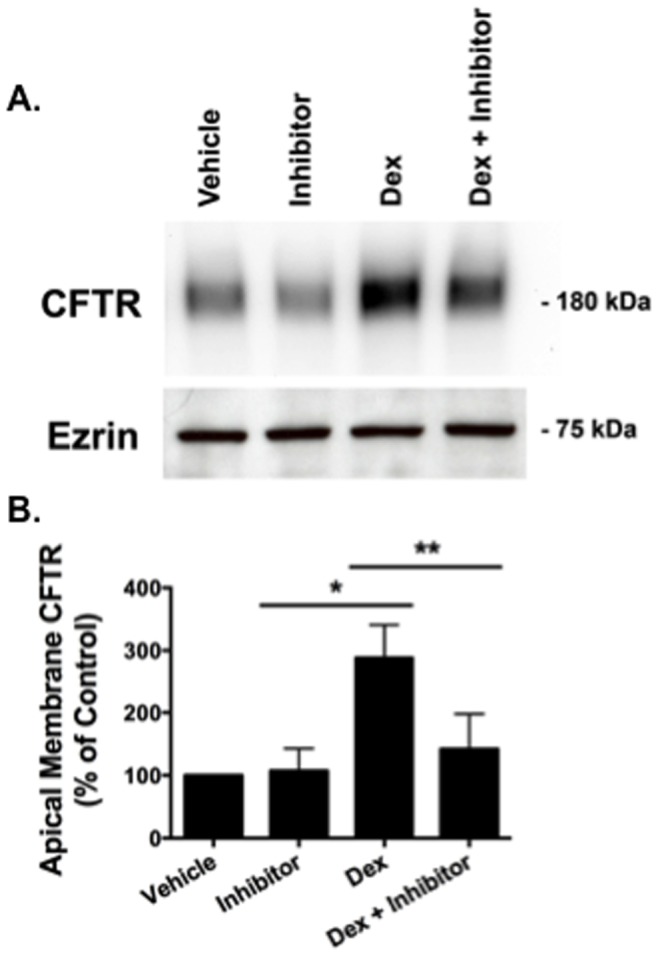
The SGK inhibitor GSK 650394 blocked the dexamethasone stimulated increase in apical membrane wt-CFTR. (A) Representative western blot, and (B) Data quantitation. CFBE cells were treated with either vehicle, GSK 650394 (100 nM), an inhibitor of SGK1, dexamethasone (50 nM), or dexamethasone (50 nM), and GSK 650394 (100 nM) for 24 hours at 37°C. The molecular mass of apical plasma membrane wt-CFTR is ∼180 kDa. Experiments performed four times. *p<0.05 versus vehicle. **p<0.05 versus dexamethasone.

**Figure 5:**
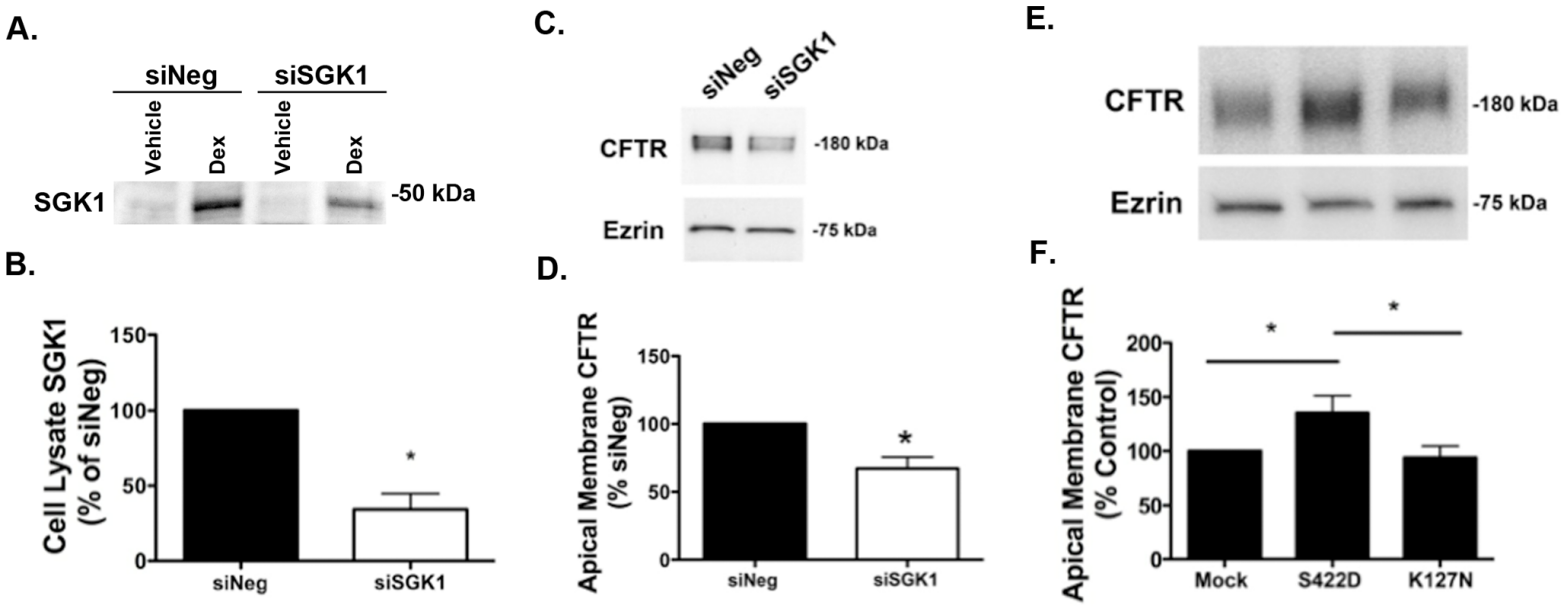
siSGK regulates apical plasma membrane wt-CFTR abundance. Representative western blots and data summaries are presented. (A) and (B), siSGK1 reduced SGK1 protein abundance in cells treated with dexamethasone. CFBE cells were transfected with siNeg or siSGK1, and 24 hours later treated with dexamethasone (50 nM) for 4 hours, whereupon SGK1 levels in cell lysates were measured by western blot analysis. (C) and (D), siSGK1 reduced wt-CFTR protein abundance in the apical membrane of cells treated with dexamethasone. CFBE cells were transfected with siNeg or siSGK1, and 24 hours later treated with dexamethasone (50 nM) for 4 hours, whereupon wt-CFTR levels in the apical plasma membrane were measured by plasma membrane protein biotinylation followed by western blot analysis as described in Methods. For A-D experiments performed three times. *p<0.05 versus siNeg. E and F. Constitutively active SGK1 (SGK1-S422D) increased apical membrane wt-CFTR, whereas a dominant negative, kinase inactive SGK1 (SGK1-K127N) had no effect on apical membrane wt-CFTR compared to mock (vector control). CFBE cells were transfected with either SGK1-S422D, SGK1-K127N, or vector control (mock). Ezrin was a loading control. Plasma membrane wt-CFTR was determined by biotinylation of apical membrane proteins as described in Methods. Experiments performed six times. *p<0.05 as indicated.

To test the hypothesis that dexamethasone increases apical membrane wt-CFTR by increasing SGK1 activity, we used an inhibitor of SGK1 kinase activity in the presence of dexamethasone. Inhibition of SGK1 kinase activity by GSK 650394 abrogated the dexamethasone induced increase in apical plasma membrane wt-CFTR, as measured by cell surface protein biotinylation and western blot analysis (Figures 4A and 4B). GSK 650394, in cells not treated with dexamethasone, had no significant effect on plasma membrane wt-CFTR (Figures 4A and 4B). To provide additional support for the observation that the dexamethasone induced increase in plasma membrane wt-CFTR is mediated by SGK1, cells were transfected with either siSGK1 or siNeg, and then the cells were treated with dexamethasone and apical membrane wt-CFTR was measured by biotinylating apical plasma membrane proteins followed by western blot analysis. Compared to siNeg, siSGK1 significantly reduced the dexamethasone induced increase in SGK1 protein abundance (Figures 5A and 5B), an effect that reduced the dexamethasone induced increase in apical plasma membrane wt-CFTR (Figures 5C and 5D). To provide additional support for the conclusion that SGK1 increases plasma membrane wt-CFTR, cells were transfected with plasmid (mock), SGK1-S422D, a constitutively active SGK1, or an inactive SGK1 (SGK1-K127N) to serve as an additional control, all in the absence of dexamethasone. Compared to mock transfected cells or cells transfected with SGK1-K127N, SGK1-422D significantly increased plasma membrane wt-CFTR (Figures 5E and 5F). Taken together the experiments in Figures 4 and 5 support the conclusion that activation of SGK1 increases the apical plasma membrane abundance of wt-CFTR in human airway epithelial cells.

### SGK1 Inhibits the Endocytic Retrieval of wt-CFTR from the Apical Plasma Membrane

**Figure 6:**
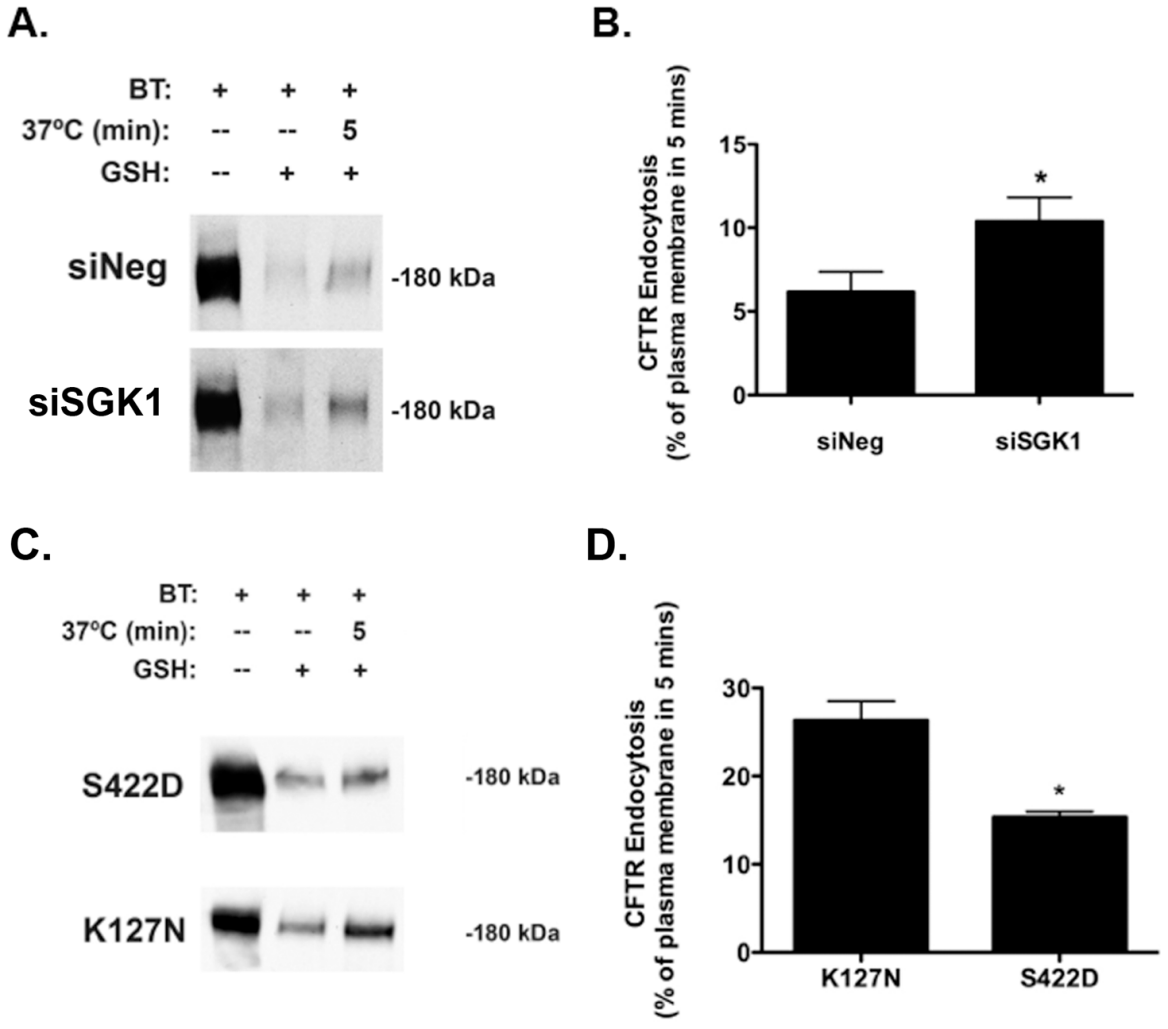
SGK1 inhibits wt-CFTR endocytosis. (A) and (B), siRNA-mediated knockdown of SGK1 in cells treated with dexamethasone (50 nM for 4 hours) enhanced the endocytic removal of wt-CFTR from the apical membrane. CFBE cells were transfected with siSGK1 or a scrambled negative control (siNeg) and 24 hours later treated with dexamethasone (50 nM) for 4 hours. (A) Representative western blot and (B) summary of data. Wt-CFTR endocytosis was measured by a method described previously in detail and in Methods [12], [22]. In brief, in three sets of cells apical membrane proteins were biotinylated at 4°C using EZ-Link™ Sulfo-NHS-SS-Biotin. Subsequently, one set of cells (lane 1) were then lysed, and the biotinylated proteins were isolated by streptavidin-agarose beads, eluted into SDS sample buffer, separated by 7.5% SDS-PAGE, and probed for wt-CFTR and ezrin. Thus, lane 1 represents the amount of wt-CFTR in the apical membrane at time = 0. A second set of cells (lane 3) were also biotinylated at 4°C and then warmed to 37°C for 5 min to allow biotinylated wt-CFTR to be endocytosed. Subsequently, the cells were cooled to 4°C, and the disulfide bonds on Sulfo-NHS-SS-biotinylated proteins remaining in the apical membrane were reduced by GSH added to the apical solution for a total of 90 min at 4°C. The cells were then lysed, and the biotinylated proteins were isolated by streptavidin-agarose beads, eluted into SDS sample buffer, separated by 7.5% SDS-PAGE, and probed for wt-CFTR and ezrin. Thus, lane 3 represent the amount of wt-CFTR in the apical membrane at time = 0 that was endocytosed in 5 minutes. Lane 2 demonstrates that GSH reduced the disulfide bonds on Sulfo-NHS-SS-biotinylated proteins in the apical membrane. Cells in this lane were biotinylated at 4°C and the disulfide bonds on Sulfo-NHS-SS-biotinylated proteins remaining in the apical membrane were reduced by GSH added to the apical solution for a total of 90 min at 4°C. The amount of biotinylated wt-CFTR remaining in the plasma membrane after GSH treatment at 4°C and without the 37°C warming was considered background (<5% compared with the amount of biotinylated wt-CFTR at 4°C without GSH treatment, i.e., lane 1) and was subtracted from the wt-CFTR biotinylated after warming to 37°C at each time point. wt-CFTR endocytosis is reported as the amount of CFTR in lane 3 (minus background) divided by the amount in lane 1 (minus background) X 100. Quantification of three experiments. *p<0.05 versus siNeg (control). (C) and (D), Constitutively active SGK (SGK1-S422D) reduced the endocytic rate of wt-CFTR from the apical membrane compared to a dominant negative SGK1 (K127N). CFBE cells were transfected with SGK1-S422D or SGK1-K127N and wt-CFTR endocytosis was measured as described above. Representative western blot (C), and summary of the data (D). Quantification of three-six experiments. *p<0.05 versus K127N.

Although others and we have demonstrated that SGK1 increases the plasma membrane abundance of wt-CFTR in a variety of cell types, the mechanism of this effect has not been elucidated. To determine if SGK1 regulates the apical membrane trafficking of wt-CFTR, studies were conducted to test the novel hypothesis that SGK1 increases apical plasma membrane wt-CFTR by inhibiting its endocytic retrieval and/or enhancing its recycling from endosomes back to the membrane. In dexamethasone treated cells siSGK1, which decreased SGK1 protein levels by ∼75% (Figures 6A and 6B) increased wt-CFTR endocytosis compared to cells transfected with siNeg (Figures 6A and 6B). By contrast, transfection of cells, not exposed to dexamethasone, with SGK1-S442D reduced wt-CFTR endocytosis compared to endocytosis of wt-CFTR in cells transfected with SGK1-K127N (Figures 6C and 6D). These experiments are consistent with the conclusion that increasing SGK1 inhibits the endocytic retrieval of wt-CFTR from the plasma membrane, which increases apical plasma membrane wt-CFTR.

### SGK1 does not Affect the Endocytic Recycling of wt-CFTR from Endosomes to the Apical Plasma Membrane

**Figure 7:**
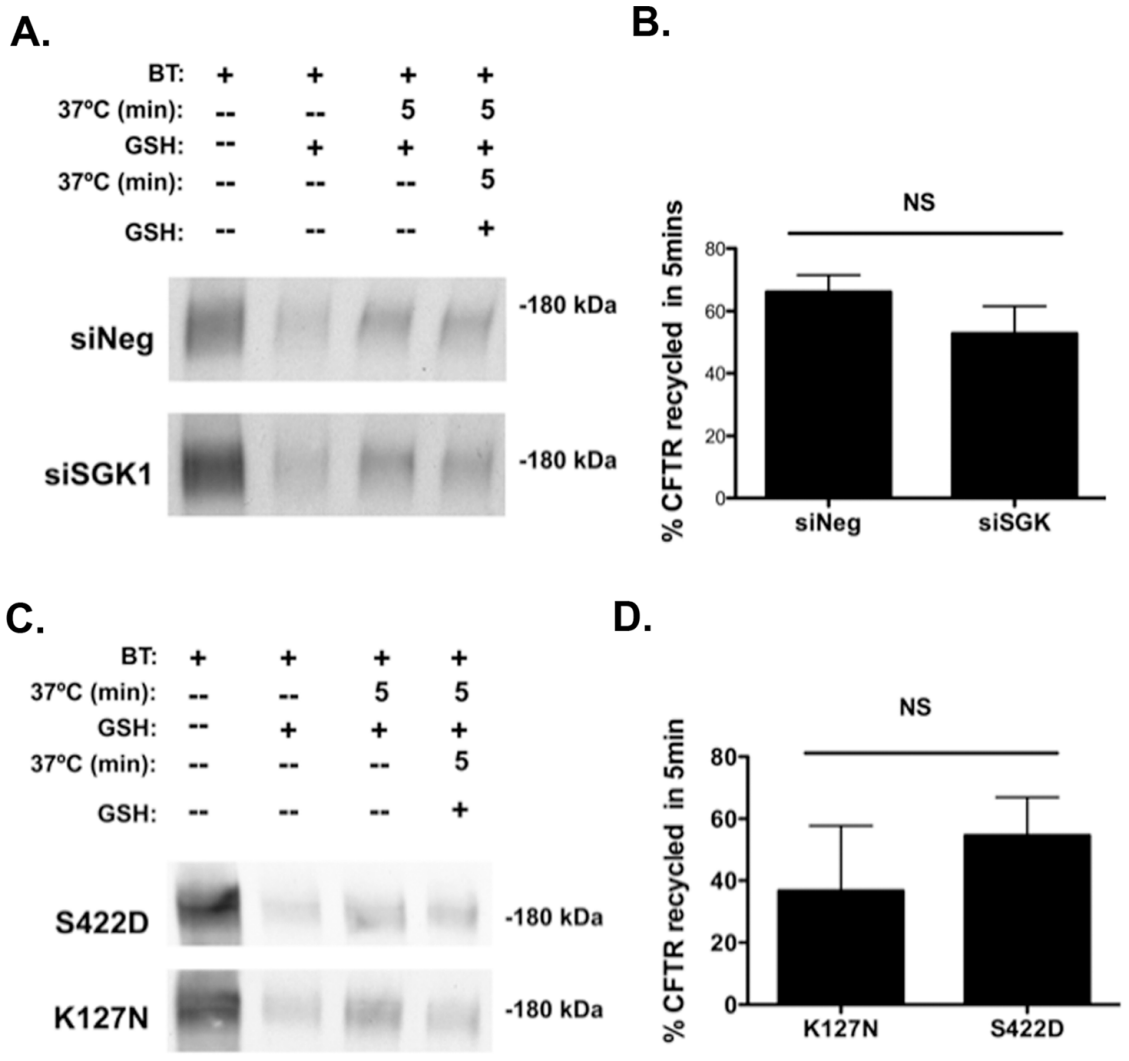
SGK1 did not alter the recycling of endocytosed wt-CFTR from endosomes back to the apical membrane. siRNA-mediated knockdown of SGK1. (A) Representative western blot, and (B) summary of the data. The recycling assay has been described in detail previously and in Methods [12], [22]. In brief, the first three lanes in A are similar to those described in Figure 6 for the endocytosis experiments. Thus, lane 1 represents the amount of wt-CFTR in the plasma membrane at time = 0, lane 3 represents the amount of wt-CFTR endocytosed in 5 minutes and lane 2 shows that GSH cleaves the disulfide bonds on Sulfo-NHS-SS-biotinylated wt-CFTR in the apical membrane. In lane 4 cells were warmed to 37°C for 5 min after biotinylation to load endocytic vesicles with biotinylated proteins. Cells were then cooled to 4°C, and the disulfide bonds on Sulfo-NHS-SS-Biotin-labeled proteins remaining in the plasma membrane were reduced by GSH at 4°C. Subsequently, cells were warmed again to 37°C for 5 min to allow endocytosed and biotinylated wt-CFTR to recycle to the plasma membrane. Cells were then cooled again to 4°C, and the disulfide bonds on biotinylated proteins in the apical membrane were reduced with GSH. The amount of recycled wt-CFTR was calculated as the difference between the amount of biotinylated wt-CFTR after the first and second GSH treatments (minus background)×100. Experiments repeated four-five times. NS, not significant. (C) and (D), The constitutively active SGK1 (SGK1-S422D) does not alter the recycling of wt-CFTR to the apical membrane. (C) Representative western blot, and (D) Summary of the data. The recycling assay was performed as described above. Experiments repeated four-five times. NS, not significant.

Figure 7 demonstrates that SGK1 does not regulate the recycling of endocytosed wt-CFTR back to the plasma membrane. In dexamethasone treated cells siSGK1, which reduced SGK1 protein levels by ∼75% (Figures 5A and 5B) had no effect on the endocytic recycling of wt-CFTR compared to cells transfected with siNeg (Figures 7A and 7B). Moreover, transfection of cells, not exposed to dexamethasone, with SGK1-S442D also had no effect on the endocytic recycling of wt-CFTR compared to cells transfected with SGK1-K127N (Figures 7C and 7D).

**Figure 8:**
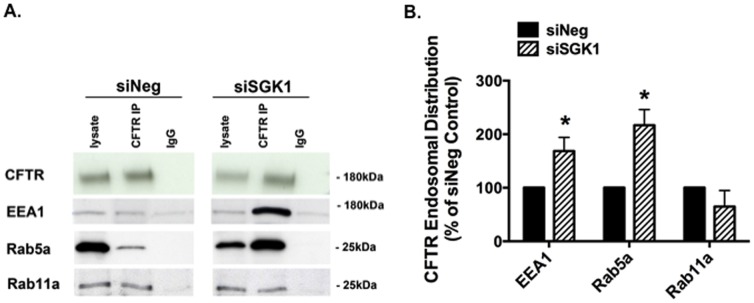
siSGK1 increased the amount of wt-CFTR in early endosomes, but not in recycling endosomes. Co-immunoprecipitation studies were conducted to determine the subcellular location of wt-CFTR in siNeg and siSGK1 transfected cells treated with dexamethasone (50 nM for 4 hours). EEA and Rab5a are markers of early endosomes, and Rab11a is a marker of recycling endosomes. wt-CFTR was immunoprecipitated, and co-immunoprecipitated proteins were eluted into SDS sample buffer, and separated by 7.5% SDS-PAGE. The blots were then probed for wt-CFTR, EEA, Rab5a, and Rab11. Co-immunoprecipitation of wt-CFTR with EEA1 and Rab5a identifies the amount of wt-CFTR in early endosomes, and co-immunoprecipitation of wt-CFTR with Rab11a identifies the amount of wt-CFTR in recycling endosomes. Quantification of data for Rab and EEA1 immunoprecipitation with wt-CFTR in siNeg and siSGK1 cells is normalized for the total amount of wt-CFTR immunoprecipitated. Blots in siNeg and siSGK1 experiments were cut for presentation, but were run one the same blot to allow for comparison. Lysate, the amount of EEA1, Rab5a Rab11 and wt-CFTR in cell lysates. CFTR IP, indicates IP with the anti-CFTR antibody and then western blot with the indicated antibody. IgG, immunoprecipitation with a non-specific antibody. (A) Representative western blots and (B) Summary of the data. Experiments repeated three times. *P<0.05 versus siNeg.

Finally, we examined the effect of siSGK1 on the subcellular location of wt-CFTR to provide additional support for the conclusion that SGK1 inhibits wt-CFTR endocytosis, but does not alter wt-CFTR recycling. To this end, cells were transfected with siNeg or siSGK1, intracellular vesicles were isolated whereupon wt-CFTR was immunoprecipitated, and the immunoprecipitated proteins were run on an SDS PAGE gel. Western blots were then performed using EEA1 and Rab5a to quantitate the amount of wt-CFTR in early endosomes, and with Rab11a, to quantitate the amount of wt-CFTR in recycling endosomes. Figures 8A and 8B demonstrate that siSGK1 increased the amount of wt-CFTR in early endosomes (i.e., the amount of co-immunoprecipitation between wt-CFTR with EEA1 and Rab5a was increased), an observation consistent with the endocytic assays demonstrating that SGK1 inhibits the endocytic removal of wt-CFTR from the apical plasma membrane into early endosomes. By contrast, siSGK1 had no effect on the amount of wt-CFTR in recycling endosomes (i.e., the amount of co-immunoprecipitation between wt-CFTR with Rab11a was unchanged), an observation consistent with the endocytic recycling assay demonstrating that SGK1 has no effect on the endocytic recycling of wt-CFTR from endosomal vesicles back to the apical plasma membrane. Taken together these experiments support the conclusion that SGK1 inhibits the endocytic retrieval of wt-CFTR from the plasma membrane, but has no effect on wt-CFTR endocytic recycling.

### SGK1 Stimulates the Endocytosis of the Epidermal Growth Factor Receptor (EGFR)

**Figure 9:**
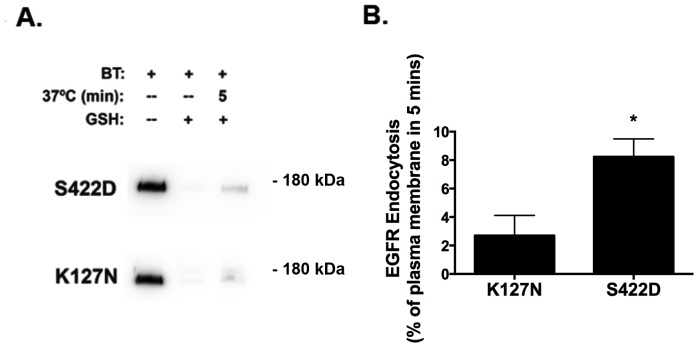
SGK1 stimulates EGFR endocytosis. (A) and (B), Constitutively active SGK1 (S422D) increased the endocytic rate of EGFR from the apical membrane compared to a dominant negative SGK1 (K127N). CFBE cells were transfected with SGK1-S422D or SGK1-K127N and EGFR endocytosis was measured as described in detail in Methods and in Figure legends above. Representative western blot (A), and summary of the data (B). Quantification of three experiments. *P<0.05 versus K127N.

The EGFR is an apical plasma membrane protein that, like wt-CFTR, is endocytosed from the apical plasma membrane of polarized epithelial cells in clathrin-coated vesicles [25], [26]. To determine if SGK1 selectively inhibited the endocytosis of wt-CFTR, or had a non-specific effect on other apical plasma membrane proteins, studies were conducted to determine if SGK1 regulates the endocytosis of the EGFR. Accordingly, polarized CFBE cells were transfected with SGK1-S422D or SGK1-K127N, and EGFR endocytosis were measured, as described in Methods. Figure 9 demonstrates that SGK1-S422D, the constitutively active form of SGK1, significantly enhanced the endocytosis of EGFR (Figures 9A and 9B) from the apical plasma membrane compared to SGK1-K127N, the inactive form of SGK1, which served as the control. This observation, taken together with studies presented above, demonstrates that SGK1 differentially regulates the endocytosis of wt-CFTR (inhibits) and the EGFR (stimulates).

## Discussion

The results of this study demonstrate for the first time that SGK1 increases the abundance of wt-CFTR in the apical plasma membrane of human airway epithelial cells by selectively inhibiting the endocytic retrieval of wt-CFTR from the plasma membrane. SGK1 also increases the amount of wt-CFTR in the plasma membrane of *Xenopus* oocytes [14], [15] and in mitochondrion rich cells in the gill of the killifish (*Fundulus heteroclitus*) [16], [17]. In a recent study Caohuy et al [18] showed that dexamethasone increases the plasma membrane expression of both wt-CFTR and ΔF508-CFTR in pancreatic cells, and that RNA interference of SGK1 blocked the stimulatory effect of dexamethasone on plasma membrane ΔF508-CFTR. Moreover, Rubenstein et al [19] reported that dexamethasone also increases wt-CFTR and ΔF508-CFTR abundance in CFBE41o- cells, and Prota et al [20] showed in Calu-3 cells that dexamethasone increases wt-CFTR biosynthesis by altering its interaction with the chaperones HSP70 and HSP90 present in the endoplasmic reticulum. The present study extends these observations and demonstrates, for the first time, that SGK1 increases plasma membrane wt-CFTR in polarized airway epithelial cells by selectively inhibiting the endocytic retrieval of wt-CFTR from the apical plasma membrane. By contrast, SGK1-S422D stimulated the endocytic retrieval of the EGRF from the apical plasma membrane.

Five lines of evidence in the present study support the conclusion that SGK1 selectively increases apical plasma membrane wt-CFTR in polarized human airway epithelial cells by inhibiting its endocytic retrieval from the membrane. First, siSGK1 increased the rate of wt-CFTR endocytosis from the plasma membrane. Second, constitutively active SGK1-S442D decreased the rate of wt-CFTR endocytosis from the membrane. Third, co-immunoprecipitation studies with markers of intracellular compartments revealed that siSGK1 increases the amount of wt-CFTR in early endosomes, but not in recycling endosomes. Fourth, neither siSGK1 nor SGK1-S442D altered the rate of wt-CFTR recycling or the amount of wt-CFTR in recycling endosomes. Fifth, SGK1-S422D increased the endocytosis of the EGFR. Thus, taken together our studies revealed for the first time in polarized airway epithelial cells that SGK1 selectively increased apical plasma membrane wt-CFTR by inhibiting its endocytic removal from the membrane.

SGK1 also regulates the plasma membrane abundance of a number of other ion channels and transport proteins including ROMK, ENaC, and Kv7.1, to name but a few, by regulating their endocytic removal from the plasma membrane [5], [13], [27]. For example, SGK1 reduces the plasma membrane abundance of ROMK, a potassium channel in the kidney, by phosphorylating WNK1, which increases the endocytic retrieval of ROMK from the plasma membrane [28]. By contrast, SGK1 increases the membrane expression of ENaC, the epithelial sodium channel, and therefore stimulates sodium reabsorption in the kidney, by phosphorylating and inhibiting Nedd4-2, an E3 ubiquitin ligase, thereby reducing the amount of ubiquitinated ENaC and inhibiting ENaC endocytosis [29]. SGK1 also increases the plasma membrane abundance of Kv7.1, a voltage gated potassium channel, by phosphorylating and inhibiting Nedd4-2-dependent endocytosis of Kv7.1 [27]. Therefore, the kinase activity of SGK1 plays a critical role in the mechanism by which SGK regulates the endocytic trafficking of other membrane proteins.

The mechanism whereby SGK1 regulates the endocytosis of wt-CFTR is not known. Caohuy et al [18] demonstrated that activation of SGK1 in pancreatic cells increased membrane ΔF508-CFTR by blocking Nedd4-2-mediated ubiquitination of ΔF508-CFTR, which reduces ΔF508-CFTR plasma membrane stability. However, in a recent study we reported that Nedd4-2 does not regulate the membrane abundance of wt-CFTR in human airway epithelial cells [30]. It is unclear if the difference in the results of these studies is due to different effects of SGK1 on wt-CFTR versus ΔF508-CFTR and/or to differences in pancreatic cells versus airway cells. Interestingly, in a recent study [19] it was shown that dexamethasone increases ΔF508-CFTR abundance in CFBE41o- cells, but did not increase the fully glycosylated, so-called C band of ΔF508-CFTR-the form that traffics to the plasma membrane, an observation consistent with the conclusion that there is an inherent difference in the effects of dexamethasone (and SGK1) on plasma membrane ΔF508-CFTR in airway cells and in pancreatic epithelial cells. Because wt-CFTR does not contain a SGK1 phosphorylation consensus sequence it is unlikely that SGK1 inhibits CFTR endocytosis in airway epithelial cells by directly phosphorylating wt-CFTR. However, it is possible that SGK1 may phosphorylate one or more of the many proteins that regulate wt-CFTR endocytosis, including, for example, c-Cbl, Dab2, Rab5, myosin VI, AP2, dynamin, and NHERF1, which interacts with the ezrin/radix/moesin (ERM) family of proteins that anchors wt-CFTR to the apical membrane by interacting with the apical actin cytoskeleton [8], [31]–[35]. In addition, SGK1 may phosphorylate a protein that may affect the membrane stability of wt-CFTR. For example, Shank2E, via its PDZ domain, binds to the C-terminus of wt-CFTR and increases plasma membrane wt-CFTR [36], [37]. Inspection of the amino acid sequence of Shank2E reveals two SGK1 consensus phosphorylation sites. Because Shank2E interacts with dynamin, a GTPase that regulates synaptic vesicle recycling, and receptor-mediated endocytosis [38], it is possible that SGK1 may phosphorylate Shank2E and inhibit wt-CFTR endocytosis. Additional studies are required to elucidate how SGK1 selectively inhibits wt-CFTR endocytosis in airway epithelial cells. Given the number of possible targets of SGK1 such an analysis will likely be a major challenge.

In this manuscript we demonstrate differential regulation of the endocytosis of additional cell surface proteins, including EGFR by SGK1. SGK1 has inverse actions on wt-CFTR and EGFR, decreasing and increasing endocytosis, respectively. To gain insight into the differential regulation by SGK1, we considered the protein-protein interactions and intracellular itineraries of wt-CFTR and EGFR. While both proteins undergo clathrin-mediated endocytosis, the protein-protein interactions and intracellular trafficking itineraries of each of these proteins are quite distinct [1], [39]. Amino acid sequence examination also reveals that neither wt-CFTR nor EGFR contain SGK1 consensus phosphorylation sequences. Thus, it is likely that SGK1 kinase activity is required for phosphorylation and regulation of proteins that in turn regulate the endocytosis of wt-CFTR and EGFR. For example, in human proximal kidney tubule cells, SGK1 overexpression has been demonstrated to increase the tyrosine phosphorylation of EGFR, an action previously demonstrated to increase endocytosis [40], [41]. In addition, SGK1-mediated activation of PIKfyve kinase leads to increased wt-CFTR channel activity and plasma membrane abundance [42]. As stated above, additional studies are required to elucidate the differential regulation of endocytic trafficking of cell plasma membrane proteins by SGK1.

Low doses of dexamethasone slow the progression of CF by reducing inflammation caused by chronic bacterial infection [43]. Because dexamethasone also increases the biosynthesis of wt-CFTR [20] as well as the plasma membrane expression of wt-CFTR [18], [20](present study), the use of dexamethasone in CF patients receiving VX-770 [44], which activates G551D-CFTR in the plasma membrane, and VX-809 [45], which increases the amount of ΔF508-CFTR in the plasma membrane, should be considered to enhance the efficacy of VX-770 and VX-809.

## Conclusion

In conclusion, our data provide the first direct evidence that, in polarized human airway epithelial cells, the glucocorticoid-induced increase in SGK1 protein abundance and activity enhances the plasma membrane abundance of wt-CFTR by inhibiting its endocytic retrieval from the plasma membrane.

## References

[1] Guggino WB, Stanton BA (2006) New insights into cystic fibrosis: molecular switches that regulate CFTR. Nature reviews Molecular cell biology 7: 426–436. 16723978 10.1038/nrm1949

[2] Cohen TS, Prince A (2012) Cystic fibrosis: a mucosal immunodeficiency syndrome. Nature medicine 18: 509–519. 10.1038/nm.2715 PMC3577071 22481418

[3] Clunes LA, Davies CM, Coakley RD, Aleksandrov AA, Henderson AG, et al (2012) Cigarette smoke exposure induces CFTR internalization and insolubility, leading to airway surface liquid dehydration. FASEB journal: official publication of the Federation of American Societies for Experimental Biology 26: 533–545. 21990373 10.1096/fj.11-192377 PMC3290447

[4] Bomberger JM, Barnaby RL, Stanton BA (2009) The Deubiquitinating Enzyme USP10 Regulates the Post-endocytic Sorting of Cystic Fibrosis Transmembrane Conductance Regulator in Airway Epithelial Cells. The Journal of biological chemistry 284: 18778–18789. 19398555 10.1074/jbc.M109.001685 PMC2707225

[5] Okiyoneda T, Barriere H, Bagdany M, Rabeh WM, Du K, et al (2010) Peripheral protein quality control removes unfolded CFTR from the plasma membrane. Science 329: 805–810. 20595578 10.1126/science.1191542 PMC5026491

[6] Cholon DM, O’Neal WK, Randell SH, Riordan JR, Gentzsch M (2010) Modulation of endocytic trafficking and apical stability of CFTR in primary human airway epithelial cultures. American journal of physiology Lung cellular and molecular physiology 298: L304–314. 20008117 10.1152/ajplung.00016.2009 PMC2838667

[7] Li C, Naren AP (2010) CFTR chloride channel in the apical compartments: spatiotemporal coupling to its interacting partners. Integrative biology: quantitative biosciences from nano to macro 2: 161–177. 20473396 10.1039/b924455g PMC2989726

[8] Ameen N, Silvis M, Bradbury NA (2007) Endocytic trafficking of CFTR in health and disease. Journal of cystic fibrosis: official journal of the European Cystic Fibrosis Society 6: 1–14. 17098482 10.1016/j.jcf.2006.09.002 PMC1964799

[9] Gentzsch M, Chang XB, Cui L, Wu Y, Ozols VV, et al (2004) Endocytic trafficking routes of wild type and ΔF508 cystic fibrosis transmembrane conductance regulator. Molecular Biology of the Cell 15: 2684–2696. 15075371 10.1091/mbc.E04-03-0176 PMC420093

[10] Swiatecka-Urban A, Brown A, Moreau-Marquis S, Renuka J, Coutermarsh B, et al (2005) The short apical membrane half-life of rescued ΔF508-cystic fibrosis transmembrane conductance regulator (CFTR) results from accelerated endocytosis of ΔF508-CFTR in polarized human airway epithelial cells. The Journal of biological chemistry 280: 36762–36772. 16131493 10.1074/jbc.M508944200

[11] Swiatecka-Urban A, Talebian L, Kanno E, Moreau-Marquis S, Coutermarsh B, et al (2007) Myosin VB is required for trafficking of CFTR in RAB11A-specific apical recycling endosomes in polarized human airway epithelial cells. The Journal of biological chemistry 282(32): 23725–36. 17462998 10.1074/jbc.M608531200

[12] Bomberger JM, Coutermarsh BA, Barnaby RL, Stanton BA (2012) Arsenic promotes ubiquitinylation and lysosomal degradation of cystic fibrosis transmembrane conductance regulator (CFTR) chloride channels in human airway epithelial cells. The Journal of biological chemistry 287: 17130–17139. 22467879 10.1074/jbc.M111.338855 PMC3366821

[13] Lang F, Shumilina E (2012) Regulation of ion channels by the serum- and glucocorticoid-inducible kinase SGK1. FASEB journal: official publication of the Federation of American Societies for Experimental Biology. 10.1096/fj.12-218230 23012321

[14] Wagner CA, Ott M, Klingel K, Beck S, Melzig J, et al (2001) Effects of the serine/threonine kinase SGK1 on the epithelial Na(+) channel (ENaC) and CFTR: implications for cystic fibrosis. Cellular physiology and biochemistry: international journal of experimental cellular physiology, biochemistry, and pharmacology 11: 209–218. 10.1159/000051935 11509829

[15] Sato JD, Chapline MC, Thibodeau R, Frizzell RA, Stanton BA (2007) Regulation of human cystic fibrosis transmembrane conductance regulator (CFTR) by serum- and glucocorticoid-inducible kinase (SGK1). Cellular and Physiological Biochemisty 20: 91–98. 10.1159/000104157 17595519

[16] Shaw JR, Sato JD, VanderHeide J, LaCasse T, Stanton CR, et al (2008) The role of SGK and CFTR in acute adaptation to seawater in fundulus heteroclitus. Cellular and Physiological Biochemisty 22: 69–78. 10.1159/000149784 18769033

[17] Shaw JR, Bomberger JM, VanderHeide J, LaCasse T, Stanton S, et al (2010) Arsenic inhibits SGK1 activation of CFTR Cl- channels in the gill of killifish, Fundulus heteroclitus. Aquatic Toxicology 98: 157–164. 20207026 10.1016/j.aquatox.2010.02.001 PMC3062055

[18] Caohuy H, Jozwik C, Pollard HB (2009) Rescue of DeltaF508-CFTR by the SGK1/Nedd4–2 signaling pathway. The Journal of biological chemistry 284: 25241–25253. 19617352 10.1074/jbc.M109.035345 PMC2757227

[19] Rubenstein RC, Lockwood SR, Lide E, Bauer R, Suaud L, et al (2011) Regulation of endogenous ENaC functional expression by CFTR and DeltaF508-CFTR in airway epithelial cells. American journal of physiology Lung cellular and molecular physiology 300: L88–L101. 20935229 10.1152/ajplung.00142.2010 PMC3023291

[20] Prota LF, Cebotaru L, Cheng J, Wright J, Vij N, et al (2012) Dexamethasone Regulates CFTR Expression in Calu-3 Cells with the Involvement of Chaperones HSP70 and HSP90. PloS one 7: e47405. 23272037 10.1371/journal.pone.0047405 PMC3521767

[21] Bebok Z, Collawn JF, Wakefield J, Parker W, Li Y, et al (2005) Failure of cAMP agonists to activate rescued deltaF508 CFTR in CFBE41o- airway epithelial monolayers. Journal of Physiology 569: 601–615. 16210354 10.1113/jphysiol.2005.096669 PMC1464253

[22] Bomberger JM, Guggino WB, Stanton BA (2011) Methods to monitor cell surface expression and endocytic trafficking of CFTR in polarized epithelial cells. Methods in molecular biology 741: 271–283. 21594791 10.1007/978-1-61779-117-8_18 PMC4402161

[23] Bomberger JM, Ye S, Maceachran DP, Koeppen K, Barnaby RL, et al (2011) A Pseudomonas aeruginosa toxin that hijacks the host ubiquitin proteolytic system. PLoS pathogens 7: e1001325. 21455491 10.1371/journal.ppat.1001325 PMC3063759

[24] Barile M, Pisitkun T, Yu MJ, Chou CL, Verbalis MJ, et al (2005) Large scale protein identification in intracellular aquaporin-2 vesicles from renal inner medullary collecting duct. Molecular and Cellular Proteomics 4: 1095–1106. 15905145 10.1074/mcp.M500049-MCP200 PMC1435688

[25] Tomas A, Futter CE, Eden ER (2013) EGF receptor trafficking: consequences for signaling and cancer. Trends in cell biology. 10.1016/j.tcb.2013.11.002 PMC3884125 24295852

[26] Weixel KM, Bradbury NA (2002) m2 binding directs the cystic fibrosis transmembrane conductance regulator to the clathrin-mediated endocytic pathway. The Journal of biological chemistry 276: 46251–44629. 10.1074/jbc.M104545200 11560923

[27] Andersen MN, Krzystanek K, Petersen F, Bomholtz SH, Olesen SP, et al.. (2013) A PI3K-SGK1 pathway promotes Kv7.1 surface expression by inhibiting Nedd4–2. The Journal of biological chemistry. 10.1074/jbc.M113.525931 PMC3873543 24214981

[28] Cheng CJ, Huang CL (2011) Activation of PI3-kinase stimulates endocytosis of ROMK via Akt1/SGK1-dependent phosphorylation of WNK1. Journal of the American Society of Nephrology: JASN 22: 460–471. 21355052 10.1681/ASN.2010060681 PMC3060440

[29] Heise CJ, Xu BE, Deaton SL, Cha SK, Cheng CJ, et al (2010) Serum and glucocorticoid-induced kinase (SGK) 1 and the epithelial sodium channel are regulated by multiple with no lysine (WNK) family members. The Journal of biological chemistry 285: 25161–25167. 20525693 10.1074/jbc.M110.103432 PMC2919078

[30] Koeppen K, Chapline C, Sato JD, Stanton BA (2012) Nedd4–2 does not regulate wt-CFTR in human airway epithelial cells. American journal of physiology Lung cellular and molecular physiology 303: L720–727. 22904170 10.1152/ajplung.00409.2011 PMC3469630

[31] Farinha CM, Matos P, Amaral MD (2013) Control of cystic fibrosis transmembrane conductance regulator membrane trafficking: not just from the endoplasmic reticulum to the Golgi. The FEBS journal 280: 4396–4406. 23773658 10.1111/febs.12392

[32] Fu L, Rab A, Tang LP, Rowe SM, Bebok Z, et al (2012) Dab2 is a key regulator of endocytosis and post-endocytic trafficking of the cystic fibrosis transmembrane conductance regulator. The Biochemical journal 441: 633–643. 21995445 10.1042/BJ20111566 PMC3646389

[33] Swiatecka-Urban A, Boyd C, Coutermarsh B, Karlson KH, Barnaby R, et al (2004) Myosin VI regulates endocytosis of the cystic fibrosis transmembrane conductance regulator. The Journal of biological chemistry 279: 38025–38031. 15247260 10.1074/jbc.M403141200

[34] Ye S, Cihil K, Stolz DB, Pilewski JM, Stanton BA, et al (2010) c-Cbl facilitates endocytosis and lysosomal degradation of cystic fibrosis transmembrane conductance regulator in human airway epithelial cells. The Journal of biological chemistry 285: 27008–27018. 20525683 10.1074/jbc.M110.139881 PMC2930700

[35] Young A, Gentzsch M, Abban CY, Jia Y, Meneses PI, et al (2009) Dynasore inhibits removal of wild-type and DeltaF508 cystic fibrosis transmembrane conductance regulator (CFTR) from the plasma membrane. The Biochemical journal 421: 377–385. 19442237 10.1042/BJ20090389

[36] Lee JH, Richter W, Namkung W, Kim KH, Kim E, et al (2007) Dynamic regulation of cystic fibrosis transmembrane conductance regulator by competitive interactions of molecular adaptors. The Journal of biological chemistry 282: 10414–10422. 17244609 10.1074/jbc.M610857200

[37] Kim JY, Han W, Namkung W, Lee JH, Kim KH, et al (2004) Inhibitory regulation of cystic fibrosis transmembrane conductance regulator anion-transporting activities by Shank2. The Journal of biological chemistry 279: 10389–10396. 14679199 10.1074/jbc.M312871200

[38] Okamoto PM, Gamby C, Wells D, Fallon J, Vallee RB (2001) Dynamin isoform-specific interaction with the shank/ProSAP scaffolding proteins of the postsynaptic density and actin cytoskeleton. The Journal of biological chemistry 276: 48458–48465. 11583995 10.1074/jbc.M104927200 PMC2715172

[39] Mayle KM, Le AM, Kamei DT (2012) The intracellular trafficking pathway of transferrin. Biochimica et biophysica acta 1820: 264–281. 21968002 10.1016/j.bbagen.2011.09.009 PMC3288267

[40] Saad S, Stevens VA, Wassef L, Poronnik P, Kelly DJ, et al (2005) High glucose transactivates the EGF receptor and up-regulates serum glucocorticoid kinase in the proximal tubule. Kidney international 68: 985–997. 16105029 10.1111/j.1523-1755.2005.00492.x

[41] Madshus IH, Stang E (2009) Internalization and intracellular sorting of the EGF receptor: a model for understanding the mechanisms of receptor trafficking. Journal of cell science 122: 3433–3439. 19759283 10.1242/jcs.050260

[42] Gehring EM, Lam RS, Siraskar G, Koutsouki E, Seebohm G, et al (2009) PIKfyve upregulates CFTR activity. Biochemical and biophysical research communications 390: 952–957. 19852935 10.1016/j.bbrc.2009.10.084

[43] Rossi L, Castro M, D’Orio F, Damonte G, Serafini S, et al (2004) Low doses of dexamethasone constantly delivered by autologous erythrocytes slow the progression of lung disease in cystic fibrosis patients. Blood cells, molecules & diseases 33: 57–63. 10.1016/j.bcmd.2004.04.004 15223012

[44] Van Goor F, Hadida S, Grootenhuis PD, Burton B, Cao D, et al (2009) Rescue of CF airway epithelial cell function in vitro by a CFTR potentiator, VX-770. Proceedings of the National Academy of Sciences of the United States of America 106: 18825–18830. 19846789 10.1073/pnas.0904709106 PMC2773991

[45] Van Goor F, Hadida S, Grootenhuis PD, Burton B, Stack JH, et al (2011) Correction of the F508del-CFTR protein processing defect in vitro by the investigational drug VX-809. Proceedings of the National Academy of Sciences of the United States of America 108: 18843–18848. 21976485 10.1073/pnas.1105787108 PMC3219147

